# Comparison of iridolenticular contact area under different light brightness between post primary acute angle closure and cataract eyes

**DOI:** 10.1186/s12886-022-02341-x

**Published:** 2022-03-11

**Authors:** Fuyao Liu, Danting Lin, Ce Zheng, Geng Wang

**Affiliations:** 1 Joint Shantou International Eye Center, Shantou University and The Chinese University of Hong Kong, Dongxia Road, Shantou, Guangdong Province The People’s Republic of China; 2 Department of Ophthalmology, Xinhua Hospital, Shanghai Jiao Tong University School of Medicine, Shanghai, The People’s Republic of China

**Keywords:** Iridolenticular contact area, Acute primary angle closure, Anterior segment optical coherence tomography

## Abstract

**Background:**

To study the iridolenticular contact area (ILCA) under different light conditions in acute primary angle closure (APAC).

**Methods:**

This cross-sectional, observational study involved 22 unilateral APAC patients and 59 cataract patients (59 eyes). Images of the APAC eyes, fellow eyes and cataract eyes were collected by anterior segment optical coherence tomography (ASOCT) under different light conditions respectively. The ILCA, anterior chamber width (ACW), anterior chamber area (ACA), lens vault (LV), angle opening distance at 750 μm (AOD750), trabecular iris space area at 750 μm (TISA750) and iris area at 750 μm (IA750) were measured using Image J software.

**Results:**

The ILCA of cataract eyes were significantly larger than APAC eyes (4.424 ± 1.208 vs 4.049 ± 2.725mm^2^, *P* = 0.034) and fellow eyes (4.424 ± 1.208 vs 3.651 ± 1.629 mm^2^, *P* = 0.008) under dark condition. Under dark condition, ILCA of APAC eyes was negatively correlated with AOD750 (*r* = -0.444, *P* = 0.038), TISA750 (*r* = -0.498, *P* = 0.018). The ILCA of cataract eyes under dark condition was significantly greater than under bright condition (4.424 ± 1.208 vs 2.526 ± 0.992 mm^2^, *P* < 0.001).

**Conclusions:**

This study showed that ILCA in both APAC eye and fellow eye were smaller than cataract eye. Future study should focus on both the contact area and force at the interface of lens and iris with larger sample size.

## Background

Glaucoma is one of the leading causes of irreversible blindness around the world [1]. Primary angle closure glaucoma (PACG) is more common in East Asia than rest of the world [1–3]. It is estimated that by 2040, the number of PACG patients in East Asia will reach 9.13 million [3]. It was reported that PACG was common among Chinese and increased the glaucoma burden in China [4]. Therefore, a full understanding of the pathogenesis of PACG is important.

It is well known that PACG eyes had shallower anterior chamber depth (ACD), smaller anterior chamber area (ACA), greater lens vault (LV) when comparing to the fellow eyes [5–7]. For acute primary angle-closure (APAC), pupil block is important pathogenesis [8]. Lowe firstly suggested that pupil block may be due to the increased area of lens-iris contact (ILC) in 1966 [9]. It was postulated that the excessive iridolenticular contact would increase the resistance of aqueous flow at the iris-lens interface and caused pupil block [10]. Nongpiur et al. confirmed that the increase of anterior surface arch may increase iridolenticular contact, which may lead to the increase of iris curvature and aggravate pupil block and anterior chamber angle crowding [5]. However, other recent studies suggested that ILC was relatively small in pupil block and decreased with pupil dilation [11, 12]. Thus, controversies exist regarding the role of iridolenticular contact in pupil block.

All previous studies measured only the iridolenticular contact distance to represent the ILC. Aptel et al. successfully used the first Pappus-Guldin centroid theorem to study 360 degree iridolenticular contact area (ILCA) in pigment dispersion syndrome with anterior segment optical coherence tomography (ASOCT) [13]. To our knowledge, no study has been carried out on the ILCA in APAC patients with ASOCT.

Therefore, the purpose of this study is to explore the changes of ILCA between APAC eyes, their fellow eyes and cataract eyes under different brightness conditions with ASOCT. The relationship between ILCA and other anterior segment parameters are also evaluated in this study.

## Methods

### Ethics approval and consent to participate

The study was designed following the ethical standards of the Declaration of Helsinki and approved by the ethical committee of Joint Shantou International Eye Center (EC20160119(1)-p01). Written informed consent was obtained from all individual participants included in the study.

### Subjects

Twenty two patients with unilateral APAC and fifty nine patients with age related cataract of Chinese ethnicity were recruited from October 2017 to March 2018. All participants underwent a complete ophthalmic examination, including a review of their medical history, measurement of best-corrected visual acuity, slit-lamp biomicroscopy, Goldmann applanation tonometry, gonioscopy and AS-OCT (CASIA SS-1000 OCT, Tomey Corporation, Nagoya, Japan). All the APAC patients had accepted topical ocular hypotensive treatment (including miotic for acute episodes) and some patients with poor intraocular pressure control had undergone anterior chamber paracentesis. If paracentesis was performed, the AS-OCT images were collected at least one day after the procedure. The inclusion criteria of the APAC patients: 1) The patient had a history of acute angle closure in one eye with at least one of the symptoms of dizziness, eye pain, nausea and vomiting. 2) Intraocular pressure exceeding 28 mmHg, conjunctival injection, corneal epithelial edema, shallow anterior chamber, middilated nonreactive pupil. 3) No history of acute attack in the other eye. The exclusion criteria: 1) secondary glaucoma such as uveitis, neovascularization and trauma. 2) History of previous glaucoma surgery (including filtering surgery, laser iridotomy, ciliary body photocoagulation, etc.). 3) Severe corneal diseases affecting gonioscopy. 4) Failure to complete AS-OCT examination. 5) Poor quality of AS-OCT images (Those in which scleral spurs could not be identified).

The inclusion criteria of the cataract patients: 1) Slit lamp microscopy showed no concurrent disease, such as pterygium, keratoconus, corneal scar, iris abnormality, etc. 2) Best corrected visual acuity (BCVA) > 0.8 or best corrected visual acuity (BCVA) > 0.8 after cataract surgery. 3) IOP < 21.0 mmHg. 4) Open anterior chamber angle. 5) No history of intraocular surgery. 6) No history of glaucoma or family history of glaucoma. The exclusion criteria: 1) The presence of eye or systemic drugs or diseases that affect light reflex. 2) Those with loose conjunctival sac or small palpebral fissure who cannot expose the limbus of the whole cornea and sclera. 3) History of intraocular surgery. 4) Inability to tolerate or cooperate with examination.

### Anterior segment OCT imaging

AS-OCT was performed using CASIA SS-1000 OCT. 3D anterior chamber angle mode in ScanType interface was selected with autofocus. Each eye was scanned under dark (approximately 0 Lux) condition and bright (approximately 100 Lux) condition respectively. Four axial images of 0–180-degree, 45–225-degree, 90–270-degree and 135–315-degree were selected and exported in JEPG format. The scleral spur was defined as the point at which a change in curvature of the inner surface of the angle wall became apparent, often presenting as an inward protrusion of the sclera. The scleral spur was marked manually by a single observer (FL). If the scleral spurs could not be identified, a second scan was performed. Images without identifiable scleral spur were excluded after the second attempt. Customized software (Anterior Segment Analysis Program [ASAP]) was used in this study to measure AS-OCT biometric parameters. ASAP is a plug-in for image processing software (ImageJ version 1.38x; public domain software, http://imagej.nih.gov/ij) [14]. After the scleral spurs was marked as reference points, ASAP automatically calculated the anatomical parameters.

Anatomical parameters include pupil diameter (PD), anterior chamber depth (ACD), anterior chamber width (ACW), anterior chamber area (ACA), lens elevation (LV), angle opening distance (AOD750), trabecular mesh iris space area (TISA750), iris area (IA750), iris curve (IC) and iridolenticular contact area (ILCA). Anterior chamber depth was defined as the distance from the corneal endothelium to the anterior surface of the lens [15]. Anterior chamber width was defined as the distance between the 2 scleral spurs. The anterior chamber area was defined as the cross-sectional area of the anterior chamber surrounded by the posterior corneal surface, the anterior iris surface and the anterior lens surface of the pupil area. The volume of anterior chamber was defined as plotting a vertical axis through the center of the ACA and rotating ACA 360 degrees around this vertical axis [16]. The AOD750, TISA750 and IA750 were measured at 750 μm from the scleral spur. The iris area was defined as the cross-sectional area of the iris between the scleral spur and the pupil area. The lens vault was defined as the vertical distance from the anterior end of lens to the horizontal line of scleral spurs on both sides [5].

### Measurement of ILCA

**Fig. 1:**
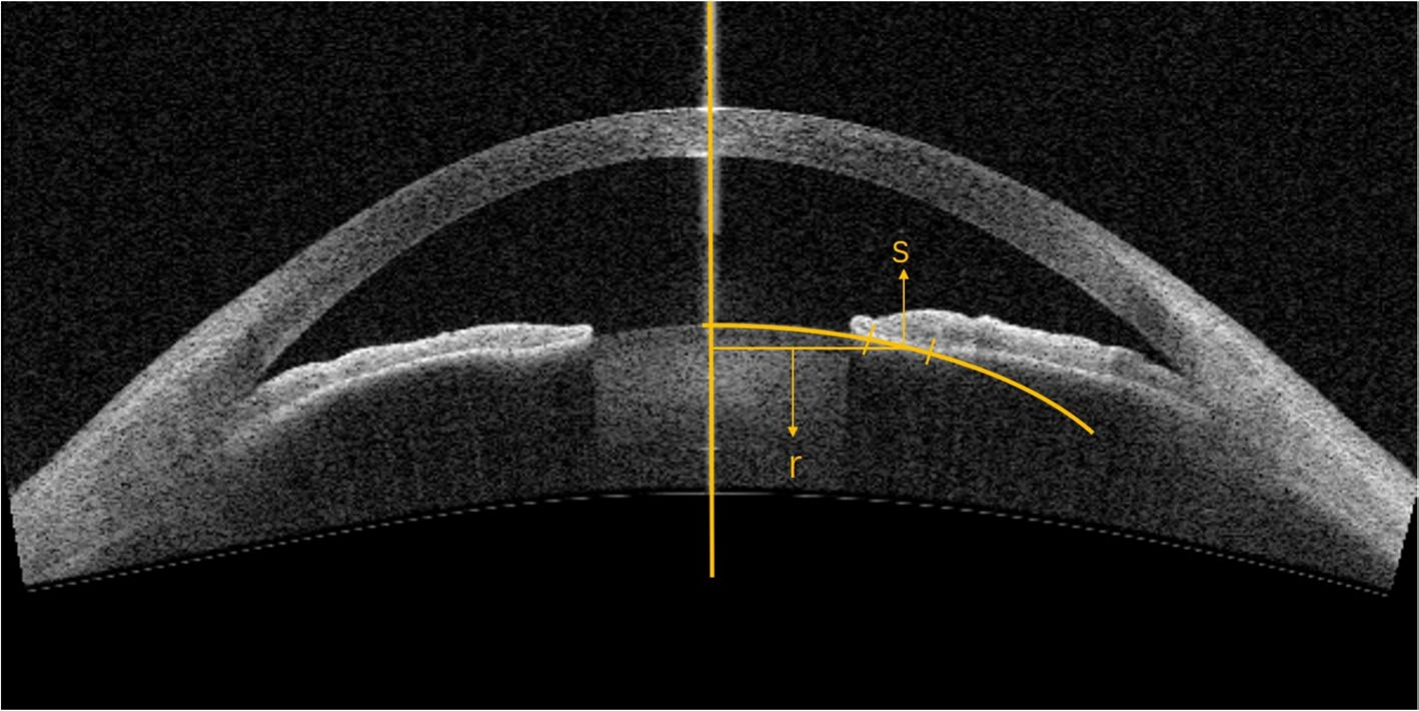
Measurement of iridolenticular contact area (ILCA): assuming that the lens scanning surface was an ellipse, the arc of lens-iris contact surface in tomographic scanning image was fitted by MatLab Image Processing Toolbox software and the length of arc was measured as S. Drawn the central axis of anterior segment. The distance between the central point of the arc and the central axis is radius r. According to the first Pappus-Guldin centroid theorem [17, 18], the contact area (A) between the full-cycle lens and the iris was calculated. The formula was A = 2πrS

The anterior segment, the iris and lens has a rotational symmetry around the antero-posterior axis. The ILCA also has a rotational symmetry around the antero-posterior axis. Assuming that the lens scanning surface was an ellipse, the arc of lens-iris contact surface in tomographic scanning image was fitted and the length of arc was measured as S. The iris and lens contact surface was defined as the area where the distance between the iris and lens outlines was less than 1 pixel in the image. Drawn the antero-posterior axis of anterior segment. The distance between the central point of the arc and the axis is radius r. According to the first Pappus-Guldin centroid theorem [17, 18], the contact area (A) between the full-cycle lens and the iris was calculated. The formula was A = 2πrS (Fig. 1). Eight cross-sectional images of the iris and lens contact surface were obtained at 0–45–90–135–180–225–270–315° meridians. The 45 degree ILCA was calculated from each cross-sectional image. Eight ILCAs from 8 cross-sectional images was added together with the following equation to get the 360 degreee ILCA:

$${A}_{360^\circ }=\sum_{i=1}^{8}2\pi riSi(45/360)$$

### Statistical analysis

Statistical analysis was performed using SPSS software version16.0 (SPSS, Inc., Chicago, IL). Differences in mean values of parametric data among eyes of different groups were examined using the independent samples Student t test. For nonparametric data, a Mann–Whitney U test was used to compare means of independent samples. Differences in mean values of ILCA of the same eye under different condition were examined using the paired samples t test. Pearson or Spearman linear correlation analysis was used to analyze the relationship between ILCA and other anterior segment parameters.

## Results

A total of 22 patients with APAC (44 eyes) and 59 patients with age-related cataract (59 eyes) were recruited in this study. All subjects were of Chinese ethnicity. The mean age was 63.09 ± 8.26(95% CI: 59.42—66.75, range: 48–79) and 65.73 ± 10.03(95% CI: 63.116—68.342, range:43–91)for APAC and cataract patients respectively. There was no significant difference in age between the APAC group and the cataract group (*P* = 0.274). But there were proportionately more females in APAC group (77.27% vs 45.76% *P* < 0.001). The median time for AS-OCT scan was 5.5 days (interquartile range, 2.5–8.5 days) after the attacks. The characteristics of the two groups were presented in Table 1.

**Table 1:** Characteristics of APACG group and cataract group

	APAC (*n* = 22)	Cataract (*n* = 59)	*P* value
Age (years, mean ± SD)^a^	63.09 ± 8.26	65.73 ± 10.03	0.274
Sex (male/female)^b^	5/17	32/27	< 0.001
eye(right/left)	/	33/26	/
Eye of acute episode (right/left)	9/13	/	/
Scanning days after attack (median, interquartile range)	5.5, 2.5–8.5	/	/

*APAC* Acute Primary Angle-Closure

^a^ Independent samples Student t test

^b^ Chi square test

The ILCA of APAC eyes and the fellow eyes (Table 2) was significantly smaller (*P* = 0.034 and *P* = 0.008 respectively) than cataract eyes under dark condition. There was no significant difference in ILCA between APAC eyes and the fellow eyes (4.049 ± 2.725 vs 3.651 ± 1.629 mm^2^, *P* = 0.742) under dark condition. Under bright condition, the ILCA was significantly greater in the fellow eyes than in cataract eyes (3.594 ± 1.631 vs 2.526 ± 0.992 mm^2^, *P* = 0.008). ILCA of cataract eyes under dark condition was significantly greater than under bright condition (4.424 ± 1.208 vs 2.526 ± 0.992 mm^2^, *P* < 0.001). No significant difference was found in ILCA of the fellow eyes between different light conditions.

**Table 2:** ILCA of different group under different light conditions

	APAC eyes	The fellow eyes	Cataract eyes
Dark (approximately 0 Lux) (mm^2^, mean ± SD)	4.049 ± 2.725	3.651 ± 1.629	4.424 ± 1.208
Bright (approximately 100 Lux) (mm^2^, mean ± SD)	/	3.594 ± 1.631	2.526 ± 0.992

*ILCA* Iridolenticular Contact Area

The pupil diameter of the fellow eyes under dark condition was significantly smaller than cataract eyes (3.587 ± 0.777 vs 4.195 ± 0.939 mm, *P* = 0.008). The APAC eyes had smaller ACW than cataract eyes (10.870 ± 0.336 vs 11.210 ± 0.510 mm, *P* = 0.005). But no significant difference in ACW was found between the fellow eyes and APAC eyes/cataract eyes. The APAC eyes had the shallowest ACD when compared with the fellow eyes (1.490 ± 0.279 vs 1.721 ± 0.139 mm, *P* = 0.002) and cataract eyes (1.490 ± 0.279 vs 2.422 ± 0.372 mm, *P* < 0.001). There was no significant difference of LV between APAC eyes and the fellow eyes (1.048 ± 0.219 vs 1.012 ± 0.202 mm, *P* = 0.549). Both APAC eyes and the fellow eyes had greater LV when compared with cataract eyes (1.048 ± 0.219 vs 0.447 ± 0.228, 1.012 ± 0.202 vs 0.447 ± 0.228 mm, both *P* < 0.001). The results of AOD750 and TISA750 were similar to those of LV. When compared to the fellow eyes, the APAC eyes had smaller IA (2.696 ± 0.504 vs 3.123 ± 0.396 mm^2^, *P* = 0.003) (Table 3).

**Table 3:** Other anterior segment parameters of different groups under dark condition (approximately 0 Lux)

Anterior segment parameters	APACG eyes (Group 1)	The fellow eyes (Group 2)	Cataract eyes (Group 3)	*P* value		
				Group 1 VS Group2	Group 1 VS Group 3	Group 2 VS Group 3
PD(mm, mean ± SD)	/	3.587 ± 0.777	4.195 ± 0.939	/	/	0.008
ACW(mm, mean ± SD)	10.870 ± 0.336	11.040 ± 0.437	11.209 ± 0.510	0.170	0.005	0.161
ACD(mm^2^, mean ± SD)	1.490 ± 0.279	1.721 ± 0.139	2.442 ± 0.366	0.002	< 0.001	< 0.001
LV(mm, mean ± SD)	1.048 ± 0.219	1.012 ± 0.202	0.447 ± 0.228	0.549	< 0.001	< 0.001
AOD750(mm, mean ± SD)	0.129 ± 0.120	0.102 ± 0.141	0.371 ± 0.141	0.186	< 0.001	< 0.001
TISA750(mm^2^, mean ± SD)	0.035 ± 0.039	0.026 ± 0.040	0.178 ± 0.075	0.243	< 0.001	< 0.001
IA(mm^2^, mean ± SD)	2.696 ± 0.504	3.123 ± 0.396	5.109 ± 11.191	0.003	< 0.001	< 0.001

Independent samples Student t test

*APACG* Acute Primary Angle-Closure Glaucoma, *PD* Pupil Diameter, *ACW* Anterior Chamber Width, *ACD* Anterior Chamber Depth, *LV* Lens Vault, *AOD 750* Angle Opening Distance at 750 μm, *TISA750* Trabecular Iris Space Area at 750 μm, *IA* Iris Area

Under bright condition, no significant difference was found between the fellow eyes and the cataract eyes (3.100 ± 0.790 vs3.111 ± 0.603 mm, *P* = 0.947) in pupil diameter. The APAC eyes had smaller ACW than cataract eyes (10.870 ± 0.330 vs 11.270 ± 0.481 mm, *P* = 0.001). But no significant difference was found between the fellow eyes and APACG eyes/cataract eyes. The results of ACD, LV, AOD750, TISA750 and IA under bright condition were just like those under dark condition (Table 4). Except for ACD, all the other anterior segment parameters including PD, ILCA, ACW, LV, AOD750 and TISA750 had significant difference under different light conditions. When under dark condition, both PD and ILCA of cataract eyes were greater than under bright condition. On the contrast, ACW, LV, AOD750 and TISA750 of cataract eyes under dark condition were smaller than under bright condition (Table 5).

**Table 4:** Other anterior segment parameters of different group under bright condition (approximately 100 Lux)

Anterior segment parameters	APACG eyes (Group 1)	The fellow eyes (Group 2)	Cataract eyes (Group 3)	*P* value		
				Group 1 VS Group2	Group 1 VS Group 3	Group 2 VS Group 3
PD(mm, mean ± SD)	/	3.100 ± 0.790	3.111 ± 0.603	/	/	0.947
ACW(mm, mean ± SD)	10.870 ± 0.330	11.050 ± 0.443	11.269 ± 0.481	0.144	0.001	0.065
ACD(mm^2^, mean ± SD)	1.500 ± 0.277	1.724 ± 0.134	2.422 ± 0.372	0.002	< 0.001	< 0.001
LV(mm, mean ± SD)	1.053 ± 0.267	1.021 ± 0.181	0.394 ± 0.228	0.659	< 0.001	< 0.001
AOD750(mm, mean ± SD)	0.137 ± 0.120	0.100 ± 0.122	0.405 ± 0.168	0.097	< 0.001	< 0.001
TISA750(mm^2^, mean ± SD)	0.035 ± 0.046	0.023 ± 0.033	0.198 ± 0.090	0.306	< 0.001	< 0.001
IA(mm^2^, mean ± SD)	2.740 ± 0.526	3.286 ± 0.313	5.098 ± 9.017	< 0.001	0.001	0.001

Independent samples Student t test

*APACG* Acute Primary Angle-Closure Glaucoma, *PD* Pupil Diameter, *ACW* Anterior Chamber Width, *ACD* Anterior Chamber Depth, *LV* Lens Vault, *AOD 750* Angle Opening Distance at 750 μm, *TISA750* Trabecular Iris Space Area at 750 μm, *IA* Iris Area

**Table 5:** Comparisons of anterior segment parameters of cataract eyes under different conditions

Anterior segment parameters	Dark (approximately 0 Lux)	Light (approximately 100 Lux)	*P* value
PD(mm, mean ± SD)	4.195 ± 0.939	3.111 ± 0.603	< 0.01
ILCA(mm^2^, mean ± SD)	4.424 ± 1.208	2.526 ± 0.992	< 0.01
ACW(mm, mean ± SD)	11.209 ± 0.510	11.269 ± 0.481	< 0.01
ACD(mm, mean ± SD)	2.442 ± 0.366	2.422 ± 0.372	0.385
LV(mm, mean ± SD)	0.394 ± 0.228	0.447 ± 0.282	0.049
AOD750(mm, mean ± SD)	0.371 ± 0.141	0.405 ± 0.168	< 0.01
TISA750(mm^2^, mean ± SD)	0.178 ± 0.075	0.198 ± 0.090	< 0.01

Paired samples t test

*PD* Pupil Diameter, *ILCA* Iridolenticular Contact Area, *ACW* Anterior Chamber Width, *ACD* Anterior Chamber Depth, *LV* Lens Vault, *AOD 750* Angle Opening Distance at 750 μm, *TISA750* Trabecular Iris Space Area at 750 μm

Pearson’s or Spearman’s linear correlation analysis was used to analyze the correlation between ILCA and other anterior segment parameters of APAC eyes under dark and bright conditions respectively. Under dark condition, the ILCA of APAC eyes were significantly negatively correlated with AOD750 (*r* = -0.444, *P* = 0.038), TISA750 (*r* = -0.498, *P* = 0.018). Under bright condition, ILCA of APAC eyes were significantly correlated with ACD (*r* = -0.608, *P* = 0.003), AOD750 (*r* = -0.451, *P* = 0.035), TISA750 (*r* = -0.450, *P* = 0.036) and LV (*r* = 0.546, *P* = 0.009). In the fellow eyes, there was no significant correlation between ILCA and other anterior segment parameters.

## Discussion

To our knowledge, this is the first study to measure ILCA in APAC patients using ASOCT. The ILCA of both APAC eyes and fellow eyes were found to be significantly smaller than cataract eyes under dark condition in current study. While under bright condition, ILCA of fellow eyes in APAC patients were significantly larger than cataract eyes. ILCA of cataract eyes under dark condition was significantly greater than under bright condition. No difference was found in fellow eyes of APAC patients under different light conditions. The AOD750 and TISA750 were found to be negatively correlated to ILCA in APAC eyes.

Current study showed that the ILCA of both APAC eye and fellow eye under dark condition was smaller than cataract eye. The old theory by Curran suggested that the excessive iridolenticular contact would increase the resistance of aqueous flow at the iris-lens interface and cause pupil block [10]. It is also suggested that the increase of the LV would lead to the increase of iridolenticular contact, which would lead to the increase of iris curvature and aggravate pupil block and crowding of anterior chamber angle [5]. However, more recent studies with new imaging techniques didn’t support the theory about iridolenticular contact in pupil block [11, 19]. Recently, Woo EK had found that for patients with pupil block, the iridolenticular contact distance under dark condition was less than that under bright condition [12]. Although Mansoori T found that the iridolenticular contact distance in patients with primary angle-closure glaucoma was greater than that in normal subjects under dark condition [19], all pupil block has been relieved by laser peripheral iridotomy in that study. A previous study also found that the iridolenticular contact distance after laser iridotomy was larger than that before laser iridotomy under both dark and bright condition [11]. These studies might just indicate that the iridolenticular contact would increase after relieve of pupil block. As the pupil block was relieved, the iris became flattened and the ILCA was increased. Since the iridolenticular contact distance was measured with only a single B scan, it was not able to provide a full view of iridolenticular contact. By measuring the 360-degree ILCA with ASOCT, current study also found that ILCA in both APAC eye and fellow eye was smaller than cataract eye, which is consistent with previous study. In contrary to the theory by Curran [10] about iridolenticular contact in pupil block, current study and previous studies [11, 19] all suggested that ILCA was smaller when pupil block occurred. In pupil block, the increase resistance at the interface between iris and lens might not be caused by excessive iridolenticular contact alone. Previous study by Zheng et al. demonstrated that the pushing force from the iris against the lens was larger in APAC eye [20]. Therefore, it should be the combination of both iris force against the lens and the iridolenticular contact that caused pupil block.

Current study showed that the ILCA was smaller in APAC eyes and fellow eyes, comparing with cataract eyes. Current study also found that ILCA of cataract eyes under dark condition was significantly greater than under bright condition. However, the ILCA of APAC eyes was increased when the AOD750 and TISA750 was decreased under both dark and light condition which indicated that the narrowing of anterior chamber angle might lead to increase of iridolenticular contact area. Since the APAC eye has narrower angle than cataract eye, the negative correlation between ILCA and angle width can only be applied in APAC eyes. The discrepant findings of ILCA between APAC and cataract eyes might be caused by the change of iris movement in APAC eyes [20]. The anterior segments were quite different between APAC and normal eyes. Current study found smaller AOD750 and higher LV in APAC eye than in cataract eye, which indicated that the iris was steeper in APAC eye than cataract eye. In normal eyes, the iris were flat (larger AOD750) and the LV was small. Although the ILCA was larger in normal eyes, there was less force from the iris against the lens. In APAC eyes, the iris was steeper (smaller AOD750) and the LV was higher. Although the ILCA was smaller, the vector force from the iris sphincter and dilator against the lens was larger [20]. As the contact area was smaller with a higher pressure, the intensity of pressure at the iris-lens interface might be even higher than control eye, which would aggravate the pupil block. Current study found a negative correlation between ILCA and angle width in APAC eyes. Previous study by Zheng et al [20]. demonstrated that the force of iris and be divided into two vector components: force against the lens and force towards the iris root. For APAC case with narrower angle, force from the iris against the lens was larger. However, normal cataract eye had wider angle and lower LV, which indicated flatter iris. As a result, there was minimal vector force against the lens in normal cataract eye with flat iris. Although the ILCA was larger, the ILCA has minimal influence on the lens in normal cataract eye. In APAC case with narrower angle, the vector force of iris against the lens was larger [20]. Current result suggested that the ILCA was increased in APAC case with narrower angle. The evidence that the iris structures of APAC eyes was different from normal eyes is mounting, including smaller IA and stronger iris sphincters. The increased ILCA might be caused by the stronger iris force against the lens in case with narrower angle. On the other hand, the increased ILCA might also increased the resistance of aqueous humor flow through pupil in APAC case with narrower angle. Since the iris was steeper in APAC eye, the larger ILCA should indicate greater iris force against the lens and higher resistance of aqueous humor at the iris-lens interface. Comparing other anterior segment parameters of APAC eyes and the fellow eyes, this study also found that the ACD, ACA and IA of APACG eyes were less than the fellow eyes under dark condition, basically similar to other studies [21–23].

Current study found that the pupil diameter of the fellow eyes under dark condition was significantly smaller than cataract eyes. Previous study by Zheng et al.suggested that iris movement was slower in angle closure eye [20]. The slower iris movement might contribute to the smaller pupil under dark condition in fellow eye of APAC. The abnormal iris movement under different light conditions is also an important reason for pupil block.

There were several limitations in current study. (1) Since the pupil was fixed and dilated in APAC eye, the pupil size would not change under different brightness. Only the ILCA under dark condition was used in current study. There was no comparison between dark and bright condition in the APAC eye. (2) Although ASOCT can capture the cross-sectional image of the whole anterior chamber in a short time, the peripheral lens surface was blocked by iris and could not be scanned by ASOCT. When calculating the ILCA, current study approximated its contact surface as a circle. The ILCA was calculated according to the shape of the iris and the curve of the anterior surface of lens. The ILCA was not measured directly by ASOCT. It might not be a totally precise measurement of the whole contact area. (3) The use of miotics would have influenced the measurements. Since all APAC eyes received miotics, no adjustment was made for the use of miotics. As the pupil was already fixed and dilated in APAC eyes, the influence of miotics on ILCA was unkown. (4) The sample size was small in current study. There were only twenty two subjects in APAC group. It was just a preliminary result about the 360-degree ILCA in APAC patients. Future study with a larger sample is warranted to clarify the role of ILCA in the pathogenesis of APAC. (5) Although the measurement of ILCA was performed automatically by software, the variability of ILCA measurement was not assessed in current study and might have influenced the results.

## Conclusions

In summary, current study showed that ILCA in both APAC eye and fellow eye were smaller than cataract eye. The APAC eyes showed smaller ACW, shallower ACD and greater LV when compared to the cataract eyes. The ILCA of APACG eyes was significantly negatively correlated with AOD750, TISA750. Excessive iridolenticular contact is unlikely to be the only cause of pupil block. Future study should focus on both the contact area and force at the interface of lens and iris with larger sample size.

## Data Availability

The excel data used to support the findings of this study is available from corresponding author upon request.

## Abbreviations

ILCA: Iridolenticular contact area
APAC: Acute primary angle closure
ASOCT: Anterior segment optical coherence tomography
ACW: Anterior chamber width
ACA: Anterior chamber area
LV: Lens vault
AOD750: Angle opening distance at 750 μm
TISA750: Trabecular iris space area at 750 μm
IA750: Iris area at 750 μm
PACG: Primary angle closure glaucoma
ACD: Anterior chamber depth
ILC: Lens-iris contact
BCVA: Best corrected visual acuity
PD: Pupil diameter
